# Linear and Non-linear Correlations Between Serum Phosphate Level and Bone Mineral Density in Type 2 Diabetes

**DOI:** 10.3389/fendo.2020.00497

**Published:** 2020-07-30

**Authors:** Yinqiu Yang, Guangwang Liu, Yao Zhang, Guiping Xu, Xilu Yi, Jing Liang, Chenhe Zhao, Jun Liang, Chao Ma, Yangli Ye, Mingxiang Yu, Xinhua Qu

**Affiliations:** ^1^Department of Endocrinology, Zhongshan Hospital, Fudan University, Shanghai, China; ^2^Department of Orthopaedics, Xuzhou Central Hospital, Xuzhou Clinical School of Xuzhou Medical University, The Affiliated Xuzhou Hospital of Medical College of Southeast University, Xuzhou Clinical Medical College of Nanjing University of Chinese Medicine, Xuzhou, China; ^3^Department of Infectious Disease, Zhongshan Hospital, Fudan University, Shanghai, China; ^4^VIP Clinical Department, Fujian Provincial Hospital, Fuzhou, China; ^5^Department of Endocrinology, Songjiang Central Hospital, Shanghai, China; ^6^Department of Endocrinology, Xuzhou Central Hospital, Xuzhou Clinical School of Xuzhou Medical University, The Affiliated Xuzhou Hospital of Medical College of Southeast University, Xuzhou Clinical Medical College of Nanjing University of Chinese Medicine, Xuzhou, China; ^7^Department of Bone and Joint Surgery, Renji Hospital, Shanghai Jiaotong University School of Medicine, Shanghai, China

**Keywords:** serum phosphate, bone mineral density, type 2 diabetes, bone metabolism, fracture risk

## Abstract

**Introduction:** Serum phosphate plays an important role in bone mineralization and might be a risk factor for many bone diseases. Patients with T2D usually have low serum phosphate level due to diet control, osmotic diuresis, and insulin stimulation. Current studies have discussed the linear association between serum phosphate and bone mineral density (BMD).

**Objective:** We aimed to analyze both the linear and non-linear correlations between serum phosphate and BMD in patients with type 2 diabetes (T2D).

**Methods:** We included 1,469 patients with T2D and obtained their basic information, laboratory measurements, and BMD data. Multivariate adjusted linear regression was used to analyze the linear associations, and we applied a two-piecewise linear regression model using a smoothing function to examine the non-linear association.

**Results:** No linear correlation was found between serum phosphate and BMD in patients with T2D. In women with T2D, we found a non-linear correlation between serum phosphate level and femur neck or total hip BMD. When serum phosphate was <1.3 mmol/L, it was positively associated with femur neck and total hip BMD, whereas when phosphate was >1.3 mmol/L, it was negatively associated with femur neck BMD.

**Conclusions:** In men with T2D, serum phosphate level was not associated with BMD. However, in women with T2D, we found a non-linear correlation between serum phosphate and femur neck or total hip BMD.

## Introduction

Over the past few years, the incidence of diabetes has increased significantly in both developed and developing countries. According to the 2018 study, the global prevalence of diabetes is about 8.8% (1). Diabetes as a chronic disease, is manifested by typical clinical polydipsia, polyuria, and more food and weight loss, and could cause many serious short-term and long-term complications, including myocardial infarction, diabetic retinopathy, neuropathy, making diabetes a growing global health concern (2). Studies have shown that in patients with type 2 diabetes (T2D), the incidence of osteoporosis and the risk of fracture are significantly increased and usually have poor prognosis (3, 4), which leads to an increased burden to the family and society. Finding the risk factors of osteoporosis in T2D is of great significance. Bone mineral density could be affected by various factors. Studies have already proved that factors including age, body mass index (BMI), smoking, alcohol use, diabetes, other endocrine diseases like parathyroid diseases, kidney function, liver function, blood pressure, Vitamin D, and growth factors levels are all the risk factors of low bone mass (5–7).

A study has shown that the serum phosphorus level was significantly lower in patients with T2D than in controls, which suggested a disorder of phosphorus metabolism in T2D (8). We speculate that there might be a correlation between this phosphorus metabolism disorder and bone metabolism disorder. In mammalian systems, phosphorus is a key element in multiple physiological processes, especially in bone mineralization (9). Phosphorus is mainly absorbed from the intestines (10) and exists in the form of inorganic phosphate (P-3) in living organisms. Serum phosphate concentration increases within hours after absorption (11). Eighty-five percent of phosphate is stored as hydroxyapatite in bones and teeth, whereas only 14 and 1% remain in intracellular and extracellular fluids, respectively, (12).

Maintenance of phosphate homeostasis is centered on the regulation of phosphate handling by the bones, intestines, and kidneys. In the skeleton, phosphate complexes with calcium and is stored as hydroxyapatite crystals, and plays an important role in bone matrix mineralization (13). The rate of bone remodeling is partly dependent on the concentration of phosphate and calcium. Without these elements, mineralization will be impaired, leading to the formation of poorly mineralized bone, which results in osteomalacia or rickets (9).

Serum phosphate seems to play a key role in bone mineralization and formation, and phosphate disorder might cause many bone diseases and fractures. In recent years, the correlation between serum phosphate level and bone mineral density (BMD) in different populations has been widely studied. A cohort study in 2017 found that serum phosphate was negatively associated with BMD in postmenopausal women, but not in men (14). Another study based on two population-based cohorts reported a negative relationship between phosphate and lumbar spine BMD in men and no association between phosphate and femur neck BMD in both sexes (15). The inverse association between serum phosphate and total body BMD in men was also found by Clarke et al. (16).

Among all the studies, only one Chinese retrospective study was conducted in patients with T2D, which showed that BMD was not correlated with serum phosphate (17). Furthermore, almost all related studies only discussed the linear correlation. Therefore, we conducted our study based on the population with T2D and aimed to clarify both the linear and non-linear correlations between serum phosphate and total lumbar, femur neck, and total hip BMDs. The null hypothesis of our study is that serum phosphate level is irrelevant to BMD.

## Materials and Methods

### Study Population

This study was a retrospective and cross-sectional study based on consecutively selected T2D patients who hospitalized in the Endocrinology Department of Zhongshan Hospital between October 2009 and January 2013. All participants were older than 18 years with definite T2D diagnosis based on the American Diabetes Association's Standards of Medical Care in Diabetes (18). They all denied recent intake of calcium tablets, diphosphonate, vitamin D, or other drugs that might influence bone metabolism or serum phosphate level. We excluded those who had heart failure, renal, or hepatic insufficiency, serious cardiovascular disease, malignant tumor, or other endocrine diseases. Disabled patients or those bed-ridden for a long period were also excluded. Our study finally included 1,469 patients with T2D (866 men and 603 women). We used individual questionnaires to determine the medical and personal history and other relevant information of each participant. Our study was approved by the ethics committee of Zhongshan Hospital, Fudan University (Approval No. B2017-172R).

### Basic Information

All participants voluntarily provided their personal and family information, and they all received a comprehensive physical examination in our hospital. We measured the blood pressure of each participant after a 30-min rest and in sitting position. We measured the height and weight of each patient in the morning after urination and before breakfast. The measurements were conducted more than once, and the average value was used. The duration of diabetes was calculated in the unit of years from the initial T2D diagnosis to the time we collected their basic information and obtained blood samples. The treatment of diabetes included oral hypoglycemic drugs, insulin injection, both hypoglycemic drugs and insulin, or none. Smoking history, alcohol intake history, disease history, and family history were defined as never or ever.

### Laboratory Measurements

During hospitalization, the serum samples of each participant were collected at 6 A.M after overnight fasting. The assays of these samples were performed within 4 h at room temperature. Serum phosphate concentration was tested by molybdenum blue method. The test sensitivity was 0.01 mmol/L and the coefficient of variation is <4.0%.

We also evaluated the blood cell counts, indexes of hepatic function, renal function, blood electrolytes, glucose, and lipid metabolism, and bone turnover markers, and others.

### BMD Measurement

According to the International Society for Clinical Densitometry guidelines, we used dual-energy X-ray absorptiometry (Hologic-Discovery, USA) to measure the BMD of each patient at three different sites: total lumbar, femur neck, and total hip.

### Statistical Analysis

In men and women with T2D, both linear and non-linear correlations between serum phosphate level and BMD were analyzed separately. Chi-square test was used to analyze categorical variables, which were expressed as numbers and proportions. Continuous variables were expressed as mean and standard deviation (SD). One-way analysis of variance was used for normally distributed continuous variables, and Kruskal-Wallis test was used for skewed continuous variables.

We used multivariate adjusted linear regression and *T*-test to analyze the linear associations between each SD in serum phosphate and BMD. We calculated the regression coefficient and corresponding 95% confidence intervals (CI). Adjusted model I was adjusted for age, body mass index (BMI), treatment for diabetes mellitus (DM), alcohol intake, smoking, and hypertension. Adjusted model II was adjusted for age, BMI, hypertension, systolic blood pressure, diastolic blood pressure, diabetes duration (years), treatment for DM, smoking, alcohol intake, family history of DM, fasting blood glucose (FBG; mmol/L), BUN (mmol/L), Cr (μmol/L), eGFR (MDRD; ml/min/1.73 m^2^), Ca (mmol/L), ALT (U/L), AST (U/L), ALP (U/L), 25(OH)D, mmol/L and PTH, pg/ml. A *P* < 0.05 was considered statistically significant.

We further examined the non-linear association between serum phosphate level and BMD. A smoothing function and a piecewise-linear regression model were applied, and we also adjusted age; treatment of DM; diabetic duration(y); smoking; drinking; family history of DM; BMI; hypertension; systolic blood pressure; diastolic blood pressure; FBG, unit; Cr, μmol/l; BUN, mmol/l; eGFR (MDRD), mL/min/1.73 m^2^; Ca, unit; ALT, U/L; AST, U/L; ALP, U/L; 25(OH)D, mmol/L, and PTH, pg/ml.

We used R packages (http://www.r-project.org) and Empower® (R) (www.empowerstats.com, X&Y Solutions Inc., Boston, MA, USA) for the statistical analyses.

## Results

### Characteristics of Participants

Our study finally included 1,469 patients with T2D (866 men and 603 women). Table 1 describes the distribution of relevant covariates. The mean age of all participants was 56.88 years, and the mean diabetes duration was 7.142 years. The mean BMD values were 0.966, 0.758, and 0.902 g/cm^2^ at total lumbar, femur neck, and total hip, respectively. We separately described the characteristics of the patients by sex and found several differences between them. The average age of the women was higher than that of the men. Diabetes duration in women was longer, but the mean HbA1c level was lower than that in men. The percentage of alcoholic drinkers and smokers in men was much higher than in women. More men have family history of diabetes, whereas more women have high blood pressure.

**Table 1:** Patient characteristics, stratified by sex.

	**Total patients (*n* = 1,469) (Mean ± SD or *N* %)**	**Male patients (*n* = 866) (Mean ± SD or *N* %)**	**Female patients (*n* = 603) (Mean ± SD or *N* %)**	***P*-value**
Age	56.880 ± 13.234	54.540 ± 13.393	60.240 ± 12.253	<0.001
Diabetic duration (years)	7.142 ± 6.886	6.089 ± 6.413	8.655 ± 7.255	<0.001
Systolic blood pressure	130.720 ± 16.314	129.521 ± 15.565	132.441 ± 17.200	<0.001
Diastolic blood pressure	80.842 ± 9.351	81.275 ± 9.279	80.221 ± 9.427	0.033
BMI	24.865 ± 3.63	24.880 ± 3.575	24.844 ± 3.719	0.853
**Laboratory findings**				
FBG, mmol/l	8.699 ± 3.051	8.721 ± 2.978	8.667 ± 3.156	0.738
HbA1C, %	9.325 ± 2.364	9.515 ± 2.408	9.055 ± 2.275	<0.001
hsCRP, mg/l	24.920 ± 8.830	4.319 ± 9.458	4.904 ± 11.201	0.296
TC, mmol/l	4.579 ± 1.082	4.573 ± 1.082	4.588 ± 1.084	0.793
TG, mmol/l	1.908 ± 1.344	1.957 ± 1.409	1.838 ± 1.241	0.097
HDL-C, mmol/l	1.114 ± 0.323	1.117 ± 0.324	1.108 ± 0.323	0.605
LDL-C, mmol/l	2.623 ± 0.908	2.614 ± 0.920	2.635 ± 0.892	0.663
BUN, mmol/l	5.868 ± 2.264	5.843 ± 2.201	5.904 ± 2.352	0.614
Cr, umol/l	69.597 ± 26.729	69.336 ± 27.435	69.972 ± 25.699	0.656
eGFR (MDRD), ml/min/1.73 m^2^	104.395 ± 39.293	93.270 ± 33.813	120.366 ± 41.090	<0.001
K, mmol/l	3.972 ± 0.383	3.976 ± 0.383	3.965 ± 0.383	0.587
Na, mmol/l	141.541 ± 3.216	141.587 ± 3.206	141.476 ± 3.232	0.516
Ca, mmol/l	2.226 ± 0.113	2.227 ± 0.116	2.225 ± 0.109	0.681
Mg, mmol/l	0.854 ± 0.085	0.853 ± 0.084	0.855 ± 0.088	0.626
TB, umol/l	10.284 ± 4.666	10.241 ± 4.508	10.346 ± 4.887	0.673
CB, umol/l	3.720 ± 1.767	3.707 ± 1.772	3.739 ± 1.762	0.732
ALT, U/L	27.093 ± 30.632	27.498 ± 29.295	26.514 ± 32.462	0.546
AST, U/L	23.658 ± 20.519	24.029 ± 21.201	23.126 ± 19.508	0.408
PTH, pg/ml	36.468 ± 14.573	36.513 ± 14.580	36.401 ± 14.577	0.888
25(OH)D, nmol/L	34.834 ± 17.058	34.384 ± 17.104	35.496 ± 16.982	0.231
**BMD, g/cm**^**2**^				
Total lumbar	0.966 ± 0.159	0.968 ± 0.156	0.964 ± 0.162	0.621
Femur neck	0.758 ± 0.132	0.759 ± 0.131	0.756 ± 0.133	0.746
Total Hip	0.902 ± 0.141	0.904 ± 0.139	0.898 ± 0.142	0.466
Treatment of diabetes				0.014
No treatment	247 (16.814%)	168 (19.400%)	79 (13.101%)	
Insulin	360 (24.506%)	200 (23.095%)	160 (26.534%)	
Oral medicine	607 (41.321%)	352 (40.647%)	255 (42.289%)	
Insulin and OM	255 (17.359%)	146 (16.859%)	109 (18.076%)	
Smoking				<0.001
Never	1,078 (73.383%)	488 (56.351%)	590 (97.844%)	
Current or ever	391 (26.617%)	378 (43.649%)	13 (2.156%)	
Drinking				<0.001
Never	1,275 (86.794%)	679 (78.406%)	596 (98.839%)	
Current or ever	194 (13.206%)	187 (21.594%)	7 (1.161%)	
Hypertension				<0.001
No	781 (53.165%)	502 (57.968%)	279 (46.269%)	
Yes	688 (46.835%)	364 (42.032%)	324 (53.731%)	
Gout or hyperuricemia				0.895
No	1,443 (98.230%)	851 (98.268%)	592 (98.176%)	
Yes	26 (1.770%)	15 (1.732%)	11 (1.824%)	
Family history of diabetes				0.044
No	878 (59.769%)	499 (57.621%)	379 (62.852%)	
Yes	591 (40.231%)	367 (42.379%)	224 (37.148%)	

*Values are mean ± SD or n (%) unless otherwise specified*.

### Linear Association Between Serum Phosphate and BMD

In the crude model, we found a positive association between serum phosphate and femur neck or total hip BMD in men with T2D (Table 2). With a 1-SD increase in serum phosphate level, femur neck BMD increased by 0.0102 g/cm^2^ (*P* = 0.045939, 95% CI = 0.0002–0.0203) and total hip BMD increased by 0.0108 g/cm^2^ (*P* = 0.047924, 95% CI = 0.0005–0.0210). However, after adjusting for age, BMI, blood pressure, diabetes duration, treatment for DM, smoking, alcohol intake, family history of DM, BUN, Cr, eGFR, FBG, Ca, ALT, AST, ALP, PTH, and 25(OH)D, this positive correlation disappeared. No association between serum phosphate level and BMD was found in the crude or multivariate-adjusted models in women.

**Table 2:** Multivariate regression for effect of serum phosphate level on total lumbar, femur neck, and total hip BMD.

**Phosphate, mmol/L per SD**	**Male patients**		**Female patients**		**Total patients**	
	**β (95% CI)**	***P***	**β (95% CI)**	***P***	**β (95% CI)**	***P***
**Total lumbar BMD**						
Crude model	0.0091 (−0.0028, 0.0211)	0.135495	−0.0064 (−0.0207, 0.0079)	0.383089	0.0024 (−0.0067, 0.0116)	0.602469
Adjusted model I	0.0082 (−0.0038, 0.0202)	0.182079	−0.0066 (−0.0213, 0.0081)	0.378642	0.0023 (−0.0070, 0.0116)	0.630558
Adjusted model II	0.0051 (−0.0082, 0.0184)	0.453812	−0.0061 (−0.0223, 0.0102)	0.465808	0.0011 (−0.0091, 0.0112)	0.837072
**Femur neck BMD**						
Crude model	0.0102 (0.0002, 0.0203)	0.045939	0.0064 (−0.0053, 0.0182)	0.284658	0.0086 (0.0010, 0.0162)	0.027405
Adjusted model I	0.0101 (−0.0001, 0.0202)	0.050471	0.0049 (−0.0072, 0.0170)	0.424954	0.0080 (0.0003, 0.0158)	0.041832
Adjusted model II	0.0047 (−0.0064, 0.0158)	0.408099	0.0037 (−0.0095, 0.0169)	0.584031	0.0043 (−0.0041, 0.0128)	0.312971
**Total hip BMD**						
Crude model	0.0108 (0.0001, 0.0215)	0.047924	0.0069 (−0.0057, 0.0194)	0.286173	0.0091 (0.0010, 0.0173)	0.028562
Adjusted model I	0.0103 (−0.0005, 0.0210)	0.061224	0.0051 (−0.0077, 0.0180)	0.434742	0.0082 (−0.0001, 0.0165)	0.050309
Adjusted model II	0.0046 (−0.0071, 0.0163)	0.440900	0.0081 (−0.0059, 0.0221)	0.259697	0.0059 (−0.0030, 0.0148)	0.191654

*Non-adjusted model adjust for: None*.

*Adjust I model adjust for: Age; BMI; Treatment of DM; Drinking; Smoking; Hypertension*.

*Adjust II model adjust for: age; treatment of DM; diabetic duration(y); smoking; drinking; family history of DM; BMI; hypertension; systolic blood pressure; diastolic blood pressure; FBG, mmol/L; Cr, umol/l; BUN, mmol/l; eGFR(MDRD), mL/min/1.73 m^2^; Ca, mmol/L; ALT, U/L; AST, U/L; ALP, U/L; 25(OH)D, mmol/L; PTH, pg/ml*.

### Non-linear Association Between Serum Phosphate and BMD

**Figure 1:**
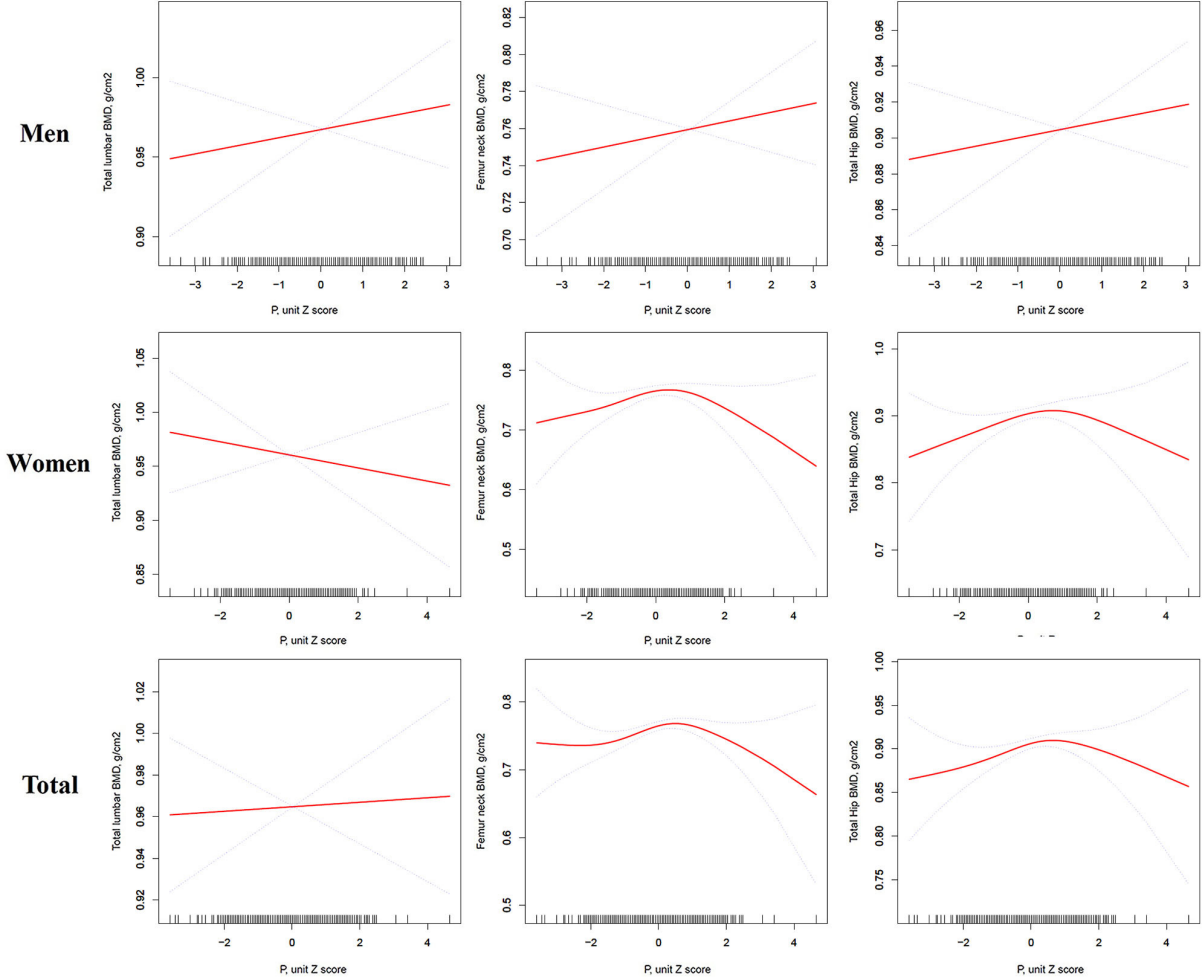
Multivariate adjusted smoothing spline plots of the total lumbar, femur neck, and total hip BMDs by serum phosphate. Red dotted lines represent the spline plots of phosphate and blue dotted lines represent the 95% confidence intervals of the spline plots. Adjusted for age; treatment of DM; diabetic duration(y); smoking; drinking; family history of DM; BMI; hypertension; systolic blood pressure; diastolic blood pressure; FBG, mmol/L; Cr, umol/l; BUN, mmol/l; eGFR(MDRD), mL/min/1.73 m^2^; Ca, mmol/L; ALT, U/L; AST, U/L; ALP, U/L; 25(OH)D, mmol/L, and PTH, pg/ml.

As shown in Figure 1, there might be some non-linear association between serum phosphate and BMD. We further applied a two-piecewise linear regression model (Table 3). Phosphate was found to be non-linearly correlated with femur neck and total hip BMD after multivariate adjustment in women. When phosphate was <1.3 mmol/L, serum phosphate was positively correlated with femur neck and total hip BMD. With a 1-SD increase in phosphate, femur neck BMD increased by 0.1259 g/cm^2^ (*P* = 0.0164, 95% CI = 0.0235–0.2282) and total hip BMD increased by 0.1303 g/cm^2^ (*P* = 0.0199, 95% CI = 0.0211–0.2395). However, when phosphate was >1.3 mmol/L, we found a negative relationship between serum phosphate level and BMD at the femur neck and total hip. A 1-SD increase in phosphate was correlated with a −0.1385 g/cm^2^ (*P* = 0.409, 95% CI = −0.2708, −0.0016) decrease in femur neck BMD. However, in men with T2D, we found no non-linear correlation between serum phosphate level and total lumbar, femur neck, or total hip BMD.

**Table 3:** Non-linear association between serum phosphate level and BMD.

**Phosphate**,	**Break**	**<K**		**>K**	
**mmol/L per SD**	**point (K)**				
		**β (95% CI)**	***p***	**β (95% CI)**	***p***
**Total lumbar BMD**					
Men	1.3	0.0234 (−0.0862, 0.1331)	0.6754	0.0273 (−0.1027, 0.1574)	0.6807
Women	1.3	−0.0393 (−0.1667, 0.0882)	0.5462	−0.0163 (−0.1808, 0.1481)	0.8456
Total	1.3	−0.0009 (−0.0832, 0.0814)	0.9829	0.0135 (−0.0871, 0.1142)	0.7921
**Femur neck BMD**					
Men	1.3	0.0712 (−0.0203, 0.1628)	0.1278	−0.0383 (−0.1470, 0.0705)	0.4904
Women	1.3	0.1259 (0.0235, 0.2282)	0.0164	−0.1385 (−0.2708, −0.0061)	0.0409
Total	1.3	0.0996 (0.0318, 0.1675)	0.0041	−0.0835 (−0.1665, −0.0004)	0.0492
**Total Hip BMD**					
Men	1.3	0.0570 (−0.0394, 0.1534)	0.2467	−0.0212 (−0.1359, 0.0934)	0.7169
Women	1.3	0.1303 (0.0211, 0.2395)	0.0199	−0.0911 (−0.2318, 0.0496)	0.2051
Total	1.3	0.0972 (0.0254, 0.1690)	0.0081	−0.0617 (−0.1496, 0.0263)	0.1696

*Adjusted for: Age; Treatment of DM; Diabetic duration(y); Smoking; Drinking; Family history of DM; BMI; Hypertension; Systolic blood pressure; Diastolic blood pressure; FBG, mmol/L; Cr, umol/l; BUN, mmol/l; eGFR(MDRD), mL/min/1.73 m^2^; Ca, mmol/L; ALT, U/L; AST, U/L; ALP, U/L; 25(OH)D, mmol/L; PTH, pg/ml*.

## Discussion/Conclusion

In this cross-sectional study, no linear correlation between serum phosphate and BMD was found both in men and women with T2D. However, our study, for the first time, reported a non-linear correlation between serum phosphate level and BMD. In women with T2D, we found a positive association between serum phosphate level and femur neck or total hip BMD with serum phosphate level <1.3 mmol/L (*P* < 0.05), but when the serum phosphate level was >1.3 mmol/L, the association became negative (*P* < 0.05). Thus, we can reject our null hypothesis. The normal range of blood phosphate is 0.90–1.34 mmol/L, according to the test standard of Zhongshan Hospital. Combined with our findings, we can conclude that maintaining a relatively high blood phosphate level, but still within the normal range, might be beneficial to BMD, whereas a serum phosphate increase above the upper limit might have a negative effect on bone health. These conclusions suggested an ideal serum phosphate level (1.3 mmol/L) for women and help us set an appropriate target value for phosphate-reducing treatment. Moreover, women with too high or too low serum phosphate should monitor their bone mineral density more frequently in order to avoid osteoporotic fractures. However, in men with T2D, no non-linear correlation was found. The different results we found between men and women patients may be partly because of the different characteristics of these two groups. The average age of the women was higher and their diabetes duration was longer, but the mean HbA1c level was lower than that in men. Smoking, drinking history, hypertension history, and family history of diabetes also have remarkable differences in men and women.

Previous studies reported a negative relationship between serum phosphate level and BMD in both postmenopausal women and men (14, 16). However, another study based on two population-based cohorts, found that such a relationship varies at different sites. According to this study, serum was only inversely related to lumbar spine BMD in men, but no relationship with the femur neck was found in either sex. This study also discussed the association between serum phosphate and fracture risk and suggested a potential threshold of serum phosphate (men: 1.1 mmol/L, women: 1.2 mmol/L), above which fracture risk was increased (15), which is consistent with the results of our study. Both animal studies and clinical studies have found an association between phosphorus intake and BMD. Koyama et al. (19) found that a Pi-high diet significantly reduced both BMD and bone mass in wild-type mice. In human subjects, phosphorus intake exceeded the recommended dosage had an independent and adverse effect on bone mass (20). A Chinese study of T2D patients reported that BMD was not correlated with serum phosphate, but this study did not analyze the possible non-linear correlation (17).

Serum phosphate level may directly affect bone metabolism. Inorganic phosphate is an essential element for the development of osteogenic cells, not only because it is an integral component of apatite crystal but also because it can affect the production rate of the bone matrix. A positive relation exists between the plasma phosphate level and the rate of skeletal growth and/or mineralization. (21) Phosphate was also essential for osteoblast differentiation and extracellular matrix mineralization and played a vital part in the mutation of growth plate and the formation of secondary ossification center (22). Thus, phosphate deprivation interfers with normal osteoblastic function, which in turn influences the process of mineralization.

Animal studies reported that calcium alone is insufficient for bone development in growing rats. At equivalent levels of calcium supplementation, the calcium phosphate salts promoted significantly greater body weight gain, femur weight, tensile strength, bone ash, bone mineralization, bone density, calcium, and phosphorus deposition, and calcium utilization (23). According to animal studies, a high-phosphate diet disturbed phosphate homeostasis, increased bone resorption, and reduced bone mineralization (19, 24, 25).

There might be many other mechanisms underlying the association between phosphate and BMD. High plasma phosphate concentrations can induce the synthesis of FGF-23 (26). FGF-23 binding to its receptor, participates in 1-hydroxylation of 25(OH)D, and induce inactivation of 1,25(OH)2D, which finally influence bone metabolism (27, 28). High-P diet-induced elevated PTH secretion leads to an increase in RANKL expression, which enhances osteoclastic bone resorption. Clinical studies also proved the positive association between PTH levels and bone turnover (29, 30) as well as the negative correlation between PTH and BMD (31). Thus, we adjusted 25(OH)D and PTH when analyzing the independent effect of serum phosphate on bone mineral density.

Compared with the general population, patients with T2D have a disorder of phosphorus metabolism. The low serum phosphate level in T2D may be due to diet control and inadequate phosphorus intake. Hyperglycemia leads to osmotic diuresis, and with the increase of urine sugar excretion, the excretion of calcium and phosphorus in the urine increases correspondingly, resulting in calcium and phosphorus loss (32, 33). Poor control of glucose level will lead to large doses of insulin and transfer of phosphorus from the extracellular compartment to the intracellular compartment, which finally leads to low serum phosphate (34). Meanwhile, low phosphorus can lead to increased insulin resistance and decreased insulin sensitivity (35, 36). As is known, the incidence of osteoporosis and the risk of fracture were significantly increased in T2D (3, 4). According to our study and other studies mentioned above, this increase may be partly explained by the disorder of phosphorus metabolism.

Our study, for the first time, reported a non-linear association between serum phosphate level and BMD. Studies proved that with a 1-SD decrease in BMD, the risk of vertebral and non-vertebral fractures increased 1.7–3 times (37, 38), which suggested BMD is an extremely useful parameter for classifying persons who are susceptible to osteoporotic fractures (39). In combination of our findings, patients maintaining an appropriate blood phosphate level might have higher BMD and lower fracture risk, while patients with too high or too low serum phosphate will be more vulnerable to osteoporotic fractures. Besides, we conducted our study in a large sample size of Asians with T2D and we adjusted for various possible confounding factors for the accuracy and reliability of the conclusion. However, our study has some limitations. First, the blood samples were collected only once, which may have caused measurement errors. Second, we used dual-energy X-ray absorptiometry to measure the BMD value, which cannot separately measure trabecular and cortical BMDs. Third, some key variables could not be further detected, because this study is cross-sectional, and this may have affected the selection of confounding factors. Some important confounding factors might have been neglected, such as dietary habits, phosphate, and other micronutrients intake, physical activity, previous fractures, FGF-23 level, and for women, the menopausal status and estrogen level, which might have also influenced our study results. Thirdly, this is a cross-sectional study, which means the causal relationship between serum phosphate levels and BMD cannot be proven. Lastly, our study only discussed the association between serum phosphate and BMD, and the association with other bone-related indicators, such as bone metabolic markers and fracture risks, still needs to be further explored in future articles.

In conclusion, in patients with T2D, serum phosphate level was not linearly correlated with BMD. However, in women with T2D, we found a non-linear correlation between serum phosphate and femur neck and total hip BMDs. When the phosphate level was <1.3 mmol/L, the serum phosphate level was positively associated with femur neck and total hip BMDs. However, when the phosphate level was >1.3 mmol/L, we found a negative correlation between serum phosphate level and BMD at femur neck.

## Data Availability Statement

The datasets generated for this study are available on request to the corresponding author.

## Ethics Statement

The studies involving human participants were reviewed and approved by Zhongshan Hospital, Fudan University. The patients/participants provided their written informed consent to participate in this study.

## Author Contributions

XQ and MY designed the research. YYa, JiL, CZ, and XQ conducted the research. GX, XY, YZ, YYe, JiL, CZ, and MY provided the essential reagents or the essential materials. YYa, GL, and XQ analyzed the data and/or performed the statistical analyses. YYa and GL wrote the manuscript. GL, JuL, CM, XQ, and MY critically revised the manuscript. All authors contributed to the article and approved the submitted version.

## Conflict of Interest

The authors declare that the research was conducted in the absence of any commercial or financial relationships that could be construed as a potential conflict of interest.
